# Epigenetic changes associated with multi-generational trauma: characterization, mechanisms, and therapeutics

**DOI:** 10.3389/fpsyt.2026.1769422

**Published:** 2026-04-01

**Authors:** Elisabeth Kac, Qian Qi, Rebecca Ryznar

**Affiliations:** 1 Rocky Vista University College of Osteopathic Medicine, Englewood, CO, United States; 2 Department of Biomedical Sciences, Rocky Vista University College of Osteopathic Medicine, Englewood, CO, United States

**Keywords:** epigenetic regulation, intergenerational trauma, parenting style, stress-response biology, transgenerational trauma, trauma therapy

## Abstract

Trauma can contribute to lasting psychological, behavioral, and physiological effects that extend across generations. Intergenerational trauma refers to trauma-related effects observed in children of exposed parents, while transgenerational trauma describes effects observed in later generations without direct exposure. Proposed mechanisms involve interacting biological and psychosocial processes, including stress-responsive regulatory systems, epigenetic variation, and caregiving environments. This review synthesizes evidence on epigenetic changes associated with acute, chronic, and complex traumatic exposures and their relevance to multi-generational outcomes. Studies published between 1990 and 2025 were identified through PubMed and Google Scholar and evaluated for reported epigenetic findings, caregiving patterns, and offspring health outcomes. Across trauma contexts, reported epigenetic variation most consistently involves pathways related to stress-response regulation, immune-inflammatory signaling, neurodevelopment, metabolic processes, and developmental programming. Patterns across exposure types suggest that acute events are most often associated with stress-related and inflammatory signaling that may influence developmental programming, whereas chronic and complex trauma reflect cumulative physiological adaptation involving broader alterations in stress-regulatory, metabolic, and neurodevelopmental systems. Offspring outcomes most consistently include increased vulnerability to anxiety, depressive symptoms, stress-related disorders, and certain chronic medical conditions, often described alongside shifts in caregiving behaviors and psychosocial environments that may shape developmental vulnerability. Interpretation of the current literature is limited by small sample sizes, varying definitions of trauma, and limited multi-generational cohorts. Overall, current evidence supports a model in which trauma-related outcomes across generations reflect interacting biological and caregiving processes, highlighting the importance of integrated molecular and psychosocial frameworks for prevention and intervention.

## 1 Introduction

Trauma can exert lasting psychological and biological effects that extend beyond directly exposed individuals and may persist across subsequent generations. Intergenerational trauma refers to trauma-related effects observed in children of exposed parents despite no direct exposure to the original event (1). Transgenerational trauma describes trauma-related effects observed in later generations that occur in the absence of direct trauma exposure in both the affected individual and their parents (2). Traumatic exposures can be broadly categorized as physical and psychological stressors, and may present as acute, chronic, or complex forms depending on the nature and duration of the exposure. Acute traumatic events involve a single overwhelming experience, chronic trauma reflects prolonged or repeated exposure, and complex trauma combines features of both, often occurring at a population level (3).

Emerging evidence suggests that these diverse forms of trauma may influence biological and psychological functioning through regulatory systems that respond to stress and environmental challenges. Epigenetic mechanisms, which modify gene expression without altering the underlying DNA sequence, have been increasingly studied as potential contributors to multi-generational patterns of vulnerability (2, 4). DNA methylation has been most frequently examined, particularly within stress-response genes of the hypothalamic-pituitary-adrenal (HPA) axis (2). In addition, histone modifications and regulation by noncoding RNAs, including microRNAs (miRNAs), transfer RNA-derived small RNAs (tsRNAs), and long noncoding RNAs (lncRNAs), have been implicated in trauma-associated gene expression differences (5, 6). Trauma-related variation has also been described in pathways relevant to neurodevelopment, immune-inflammatory signaling, circadian regulation, metabolism, and memory formation (7–9).

Developmental timing appears to play an important role in shaping outcomes, as exposures occurring *in utero* or during early childhood, periods characterized by heightened biological plasticity, have been associated with more pronounced neurocognitive and psychological effects (7, 10, 11). Alterations across stress-regulatory, neurodevelopmental, immune, and metabolic systems have been linked to increased vulnerability to post-traumatic stress disorder (PTSD), depression, anxiety, suicide, and chronic medical conditions, including metabolic syndrome and immune dysregulation (12).

A range of biological samples and analytic approaches have been used to investigate trauma-associated epigenetic variation across generations. Blood, saliva, and urine are most commonly used in human studies given their feasibility and accessibility (6). Sequencing-based approaches remain the gold standard for DNA methylation profiling, evolving from reduced representation bisulfite sequencing (RRBS) to enzymatic methyl-seq and newer TET-assisted methods (13, 14). Third-generation long-read sequencing technologies provide opportunities for direct detection of methylation and improved resolution of complex genomic regions (13). Methods for profiling RNA modifications are less established but continue to develop, with approaches such as m6A sequencing, pseudouridine sequencing, and specialized techniques including PANDORA-seq and cP-RNA-seq expanding the capacity to characterize small noncoding RNAs (14–16).

Psychosocial and caregiving environments may further shape how trauma-related biological differences are expressed across generations. Dysfunctional parenting styles encompass patterns such as overprotection, abuse, indifference, inconsistent discipline, and emotional withdrawal (17, 18). Parents with trauma histories may demonstrate disrupted caregiving behaviors, which have been associated with adverse psychological and physiological development in offspring (18). Interventions including psychological support and family-based educational programs have shown promise in mitigating intergenerational effects (19). However, the extent to which such interventions influence underlying epigenetic regulation remains incompletely understood.

**Figure 1:**
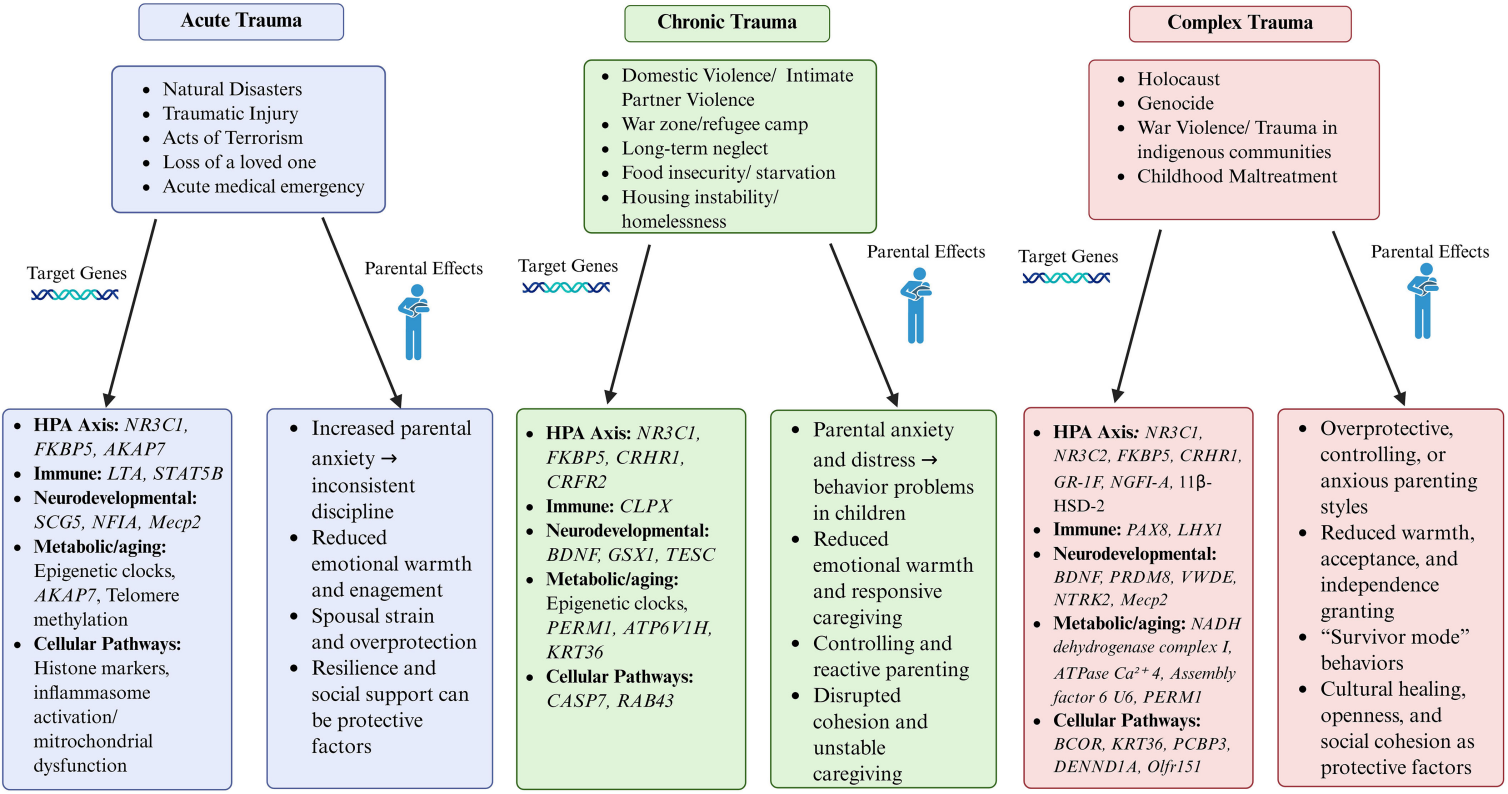
Biological and parenting pathways underlying multi-generational trauma. Acute, chronic, and complex forms of trauma influence biological and psychosocial transmission pathways across generations. Each trauma type is associated with alterations in the HPA axis, immune, neurodevelopmental, metabolic, and cellular regulation, as well as disruptions in parenting behavior. Supportive caregiving, emotional availability, and social resilience can mitigate the transmission of trauma-related risk.

This review synthesizes evidence on epigenetic changes associated with acute, chronic, and complex traumatic events, highlighting implicated molecular mechanisms and examining their relevance to multi-generational outcomes. By integrating biological and psychosocial perspectives, this work seeks to clarify emerging patterns of trauma-associated biological embedding and identify potential avenues for prevention and intervention (**Figure 1**).

## 2 Acute trauma

Across different forms of acute trauma, brief but severe stress appears to be associated with lasting biological and physiological changes. Evidence from multiple trauma models points to shared involvement of stress-response signaling, immune-inflammatory activity, and neurodevelopmental processes (10, 11, 20–53). When exposures occur during pregnancy, maternal physiological and psychological responses may influence the intrauterine environment, with potential implications for fetal developmental programming. The timing of exposure, overall stress burden, and maternal perception of the event appear to shape the development of these biological responses. In addition to biological pathways, post-trauma caregiving environments may modify how stress-related vulnerability is expressed.

### 2.1 Natural disasters

Natural disasters provide a well-studied model for understanding how acute environmental stress may become biologically embedded across generations. Findings across these settings implicate interacting biological systems, particularly neurodevelopmental pathways, immune and metabolic regulation, and stress-response signaling (10, 11, 20–28). Prenatal timing appears especially influential, while exposure severity and maternal psychological response further modify downstream biological effects.

Developing neural systems appear particularly sensitive to prenatal disaster exposure. Early-life environmental stress has been associated with differences in brain maturation and cognitive development during critical developmental windows. Maternal stress during the Quebec ice storm was linked to variation in brain structure and cognitive performance, particularly in regions involved in emotional regulation and executive functioning (11). Greater exposure severity was associated with lower childhood IQ and language performance (20). At the molecular level, prenatal exposure has been linked to DNA methylation differences in genes related to neuroendocrine signaling, including *SCG5* (21). Similar developmental sensitivity has been reported in earthquake cohorts, where early gestational exposure was associated with poorer working memory in adulthood (10). The magnitude of these effects appears to reflect interaction between objective exposure severity and maternal subjective perception, suggesting coordinated influence of physiological and environmental signaling (22).

Beyond neural outcomes, prenatal disaster exposure has been linked to immune and metabolic regulatory changes. Drought-related prenatal stress has been associated with differential DNA methylation in genes involved in metabolic, immune, and stress-regulatory pathways (23). These patterns correspond with reduced early growth, consistent with altered metabolic and endocrine signaling during development (23). Prenatal disaster exposure has been associated with accelerated epigenetic aging and shorter methylation-based telomeres, markers linked to increased chronic disease risk and immune decline (24). Additional findings from the Quebec ice storm cohort demonstrate methylation differences of immune-related genes such as *LTA*, supporting involvement of inflammatory regulation (21).

Stress-response regulation represents another pathway influenced by prenatal disaster exposure. Gestational exposure to the Tangshan earthquake was associated with increased methylation of *NR3C1*, a key regulator of glucocorticoid signaling (10). Individuals exposed prenatally, particularly early in gestation, showed higher rates of depressive symptoms compared with those exposed postnatally or not at all (25).

Psychosocial and caregiving environments further influence developmental expression. Increased parental stress has been associated with reduced caregiving consistency, emotional availability, and responsiveness (26, 27). These caregiving differences correspond with increased emotional and behavioral difficulties in children (26, 27). More supportive and structured caregiving appears protective, whereas inconsistent or disrupted caregiving is associated with greater behavioral challenges (27). Variation in caregiving has also been linked to differences in neural processing within reward and threat-related systems (28).

Natural disaster models suggest that acute gestational stress involves coordinated changes across neurodevelopmental, immune-metabolic, and stress-regulatory systems. Variation in maternal psychological response and post-disaster caregiving further influences how these biological alterations translate into developmental outcomes.

### 2.2 Traumatic injury

Acute physical trauma represents a physiological stressor capable of initiating systemic biological responses extending beyond the injured individual. Across injury contexts, research demonstrates coordinated activation of immune-inflammatory signaling, neuroendocrine regulation, and epigenetic changes (29–34). When injury occurs during pregnancy, these systemic responses may influence the intrauterine environment through maternal physiological adaptation. Pregnancy itself is a biologically dynamic state, and emerging evidence suggests stress-related signaling during this period may affect both maternal and fetal processes (35).

Severe injury has been associated with widespread epigenetic alterations, particularly in inflammatory pathways. Persistent methylation differences have been observed in regulatory regions involved in inflammation and coagulation (29). Similar epigenetic changes have been reported following traumatic brain injury, including alterations in DNA methylation, histone regulation, and mitochondrial signaling, processes involved in inflammation, neuroplasticity, and recovery (30). Comparable inflammatory interactions have also been described following spinal cord injury (31). Although direct evidence linking maternal injury to fetal epigenetic outcomes remains limited, systemic immune and hormonal responses following trauma provide a possible biological pathway through which maternal physiological disruption may alter intrauterine developmental signaling.

Psychosocial factors also contribute to developmental trajectories. Neurological injury and chronic pain have been associated with reduced emotional availability, lower parental engagement, and inconsistent caregiving, patterns linked to increased emotional and behavioral difficulties in children (32, 33). In contrast, contexts characterized by strong social support and adaptive coping demonstrate more stable caregiving and developmental outcomes, comparable to those of non-injured households (34).

Traumatic injury models suggest that acute physiological trauma is associated with coordinated immune-inflammatory, neuroendocrine, and epigenetic changes. Although direct fetal evidence remains limited, systemic inflammatory and hormonal responses following maternal injury provide a possible pathway through which intrauterine signaling may be altered. Differences in caregiving stability and social support further modify developmental outcomes.

### 2.3 Acts of terrorism

Terrorist attacks represent acute psychological stressors associated with biological and emotional effects. Findings across terrorism-related exposures implicate stress-response regulation, immune signaling, and neurodevelopmental pathways (36–43). When exposure occurs during pregnancy, maternal stress physiology may influence fetal development through hormonal and epigenetic mechanisms.

Alterations in stress-response regulation appear central in terrorism-related models. Following the September 11 attacks, infants born to mothers with PTSD who were directly exposed to the World Trade Center demonstrated lower baseline salivary cortisol levels, particularly with third-trimester exposure (36). Maternal cortisol levels were associated with infant behavioral responses to novelty (37). Reduced expression of *FKBP5*, a regulator of glucocorticoid signaling, has also been reported following prenatal psychological stress (38).

Immune and neurodevelopmental pathways may also be involved in responses to terrorism-related stress. PTSD following terrorism exposure has also been associated with altered expression of immune-regulatory genes such as *STAT5B*, supporting interaction between psychological stress and cytokine signaling pathways (39). Altered expression of genes involved in neural development and emotional regulation, including *NFIA*, has also been reported (39). At the population level, prenatal terrorism exposure has been associated with increased schizophrenia risk and lower birth weight, further suggesting sensitivity of fetal neurodevelopment and growth to maternal stress physiology (40, 41).

Caregiving context further influences how biological responses translate into developmental outcomes. Maternal PTSD and depression have been associated with increased emotional and behavioral vulnerability in children, including emotional reactivity and aggression (42). In contrast, warm and responsive caregiving environments correspond with fewer psychological symptoms, whereas less responsive parenting is linked to worse behavioral outcomes (43).

Terrorism-related models suggest that acute psychological stress may involve coordinated alterations in stress-response regulation, immune signaling, neurodevelopmental processes, and psychosocial functioning. Maternal psychological functioning and caregiving environments appear to interact with these biological changes, shaping how vulnerability is expressed over time.

### 2.4 Sudden loss of a loved one

The sudden death of a close family member or partner during pregnancy represents an intense emotional stressor capable of producing sustained physiological effects. Research across bereavement contexts implicates stress-response signaling, immune-inflammatory activation, and biological aging processes (44–48).

Population and longitudinal studies report associations between bereavement and accelerated epigenetic aging, particularly in markers reflecting inflammatory and metabolic burden (44). Individuals exposed to repeated major losses demonstrate faster progression on DNA methylation-based aging clocks such as PhenoAge, GrimAge, and DunedinPACE (44). These findings align with chronic inflammatory activation and cumulative physiological strain.

Grief exposure has been linked to dysregulated HPA axis activity, altered glucocorticoid feedback, and increased pro-inflammatory cytokine signaling, alongside downstream changes in gene expression (45). Multigenerational cohort data indicate parental bereavement corresponds with immune-related outcomes in offspring, including increased risk of asthma, allergic disease, and autoimmune conditions (45). The consistent association between inflammatory activation and accelerated epigenetic aging suggests cumulative physiological load may represent a key pathway linking bereavement to long-term health trajectories.

Epigenetic variation has also been observed in pathways involving oxytocin and dopamine signaling, systems central to attachment, motivation, and emotional regulation (46). Methylation differences within these pathways have been associated with variation in emotional flexibility, social engagement, and adaptive coping (46). Rather than reflecting uniformly maladaptive changes, these findings suggest that biological responses to early adversity may involve changes in neuroregulatory systems, with behavioral expression shaped by interacting environmental and developmental factors.

Caregiver psychological functioning following loss influences emotional availability, warmth, and consistency in parenting (47). Higher caregiver self-regulation and adaptive coping are associated with fewer emotional and behavioral difficulties in children (48). In contrast, persistent or complicated grief has been linked to withdrawal and increased child distress (48).

Bereavement models suggest that acute emotional trauma is associated with coordinated changes spanning stress-response regulation, immune-inflammatory signaling, biological aging, neurobehavioral regulation, and psychosocial environments. Caregiving stability and adaptive coping further appear to modify how these biological responses translate into developmental outcomes.

### 2.5 Acute medical emergencies

Severe medical complications during pregnancy represent major physiological stressors capable of altering maternal and fetal biology. Across models of acute maternal illness, evidence points toward involvement of immune-inflammatory signaling, endocrine and metabolic regulation, and epigenetic remodeling (49–53).

Experimental models of maternal stroke demonstrate widespread epigenetic alterations, including histone methylation changes linked to oxidative stress, mitochondrial dysfunction, and immune activation (49). These changes correspond with increased inflammasome signaling and downstream immune regulation (49). Maternal immune activation models similarly demonstrate global DNA methylation differences affecting genes involved in synaptic development and neural plasticity, suggesting inflammation-mediated signaling may influence fetal development (50).

Human pregnancy complications such as gestational hypertension, gestational diabetes, and preeclampsia have been associated with altered DNA methylation patterns in maternal and fetal tissues, particularly in pathways related to placental function, vascular regulation, and fetal growth (51). Differences in placental methylation and inflammatory signaling across pregnancy complications suggest disruption of coordinated maternal-placental regulation may represent a primary mechanism influencing fetal developmental programming (52).

Severe maternal morbidity and medical crises have been associated with delayed bonding and early caregiving disruption, including reduced physical proximity and difficulty initiating breastfeeding (53). These early relational disruptions may influence stress regulation and developmental outcomes in offspring.

Models of acute medical emergencies suggest that severe physiological stress during pregnancy may involve coordinated alterations across immune-inflammatory signaling, endocrine and metabolic regulation, epigenetic processes, and early caregiving environments. The magnitude and persistence of these effects appear to depend on developmental timing, severity of maternal physiological disruption, and the stability of the postnatal caregiving context.

When considered together, acute trauma models suggest brief but severe stress exposures engage multiple biological systems, with developmental impact shaped by exposure timing, physiological response, and post-trauma caregiving context (**Tables 1**, **2**).

**Table 1:** Epigenetic mechanisms linking acute trauma to offspring development.

Exposure/population	Sample type	Epigenetic changes	Consequences
Natural disasters			
Quebec ice storm (104 prenatally exposed)	MRI/fMRI	NM	Brain structure/connectivity changes → ↑ psychiatric disorder risk (11)
Quebec ice storm (89 prenatally exposed)	Neurocognitive testing	NM	↓ IQ, ↓ language scores w/ ↑ maternal stress (20)
Quebec ice storm (36 prenatally exposed)	Blood, saliva	↑ *SCG5*, ↑ *LTA* methylation	Altered cognition; immune dysregulation (21)
Quebec ice storm (34 mother appraisal)	Blood	>1,500 DNA methylation differences	Negative appraisal → ↑ methylation, ↑ vulnerability (22)
Drought (213 prenatally exposed)	Blood	16 CpG including *AKAP7*	↓ BW mediated by *AKAP7* methylation (23)
Drought (104 exposed vs. 104 sibling control)	Saliva	Epigenetic age acceleration; telomere shortening	↑ chronic disease risk (24)
Earthquake (176 exposed individuals)	Blood	↑ *NR3C1* methylation	↓ working memory (10)
Earthquake (1,328 various exposures)	Psychological assessment	NM	↑ depression, ↑ suicidal ideation (prenatal exposure) (25)
Traumatic injury			
Trauma vs. elective surgery (60 vs. 57)	Blood	>10,000 CpGs, >1,000 DMRs	Trauma-induced immune dysregulation (29)
Acts of terrorism			
9/11 (38 mothers and infants)	Saliva	NM	↓ infant cortisol w/3rd trimester maternal exposure (36)
9/11 (98 pregnant women)	Saliva, surveys	NM	↓ maternal cortisol, infant's ↑ distress, ↓ adaptability (37)
9/11 (35 adults, 15 w/ PTSD)	Blood	↓ *FKBP5*	Altered HPA axis and immune function (38)
9/11 (40 adults w/ and w/out PTSD)	Blood	↓ *STAT5B* and ↓ *NFIA*	Dysregulated stress response, ↑ PTSD risk (39)
Terror exposure (201,048 Israeli births)	Population registry	NM	2x ↑ risk of schizophrenia (40)
Landmine explosions (781,000 Colombian births)	Birth records	NM	↓ BW (8.7 g less than siblings) (41)
Loss of a loved one			
Familial loss (3,963 U.S. participants)	Blood	Epigenetic age acceleration	↑ chronic disease risk (44)
Familial loss (3 generations, Sweden)	Population registry	HPA/immune disruption	Maternal → ↑ asthma/ allergy; paternal→ ↑ autoimmune risk (45)
Familial loss (371 participants)	Blood	Methylated oxytocin/ dopamine activity	↑ resilience and openness, ↓ attachment avoidance (46)
Acute medical emergencies			
Pregnancy complication (50 women)	Placenta, umbilical cord blood	↓ placental methylation in GDM/preeclampsia, ↑ in obesity	Altered fetal growth (↓ length, head circumference) (52)

↑, increase; ↓, decrease; NM, not measured; BW, birth weight; CpG, cytosine-phosphate- guanine site; DMR, differentially methylated region; GDM, gestational diabetes mellitus; CVD, cardiovascular disease.

This table summarizes studies examining how acute trauma exposures influence offspring development through epigenetic pathways. The developmental window for each study (prenatal, perinatal, or early childhood) is included, given its importance for interpreting trauma-related epigenetic changes. Reported findings include alterations in DNA methylation and other regulatory pathways involved in stress physiology, immune signaling, and neurodevelopment. Such alterations are associated with changes in cognition, emotional regulation, and increased vulnerability to mental and physical health conditions. Select studies without direct epigenetic measurements were included when offspring outcomes have been independently linked to trauma-associated epigenetic mechanisms in related populations.

**Table 2:** Parenting and offspring consequences of acute trauma.

Exposure/ population	Method	Parenting factors	Child outcomes
Natural disasters			
Hurricane Harvey (140 exposed parents)	Parent surveys; child questionnaires	↑ parental anxiety, inconsistent discipline, resource loss	↑ emotional/behavioral problems, hyperactivity (26)
Turkiye Earthquake (358 preschoolers)	Parent surveys; child behavior ratings	↑ parental distress	↑ psychiatric problems, ↓ prosocial behavior (27)
Hurricane Sandy (74 children)	EEG tasks, questionnaires	Parenting styles (promotion vs prevention)	Low promotion → ↓ reward response; high prevention → ↑ threat sensitivity (28)
Traumatic injury			
TBI families (32 families; 1 parent with TBI vs. controls)	Interviews, questionnaires	↓ parental goal-setting, warmth, involvement	Children ↑ depressive sx, ↓ consistency (53)
Spinal cord injury (62 families)	Phone interviews; questionnaires	No significant difference in warmth, structure, or strictness	No significant difference in child behavior (34)
Acts of terrorism			
9/11 (116 preschool children)	Maternal/teacher behavioral ratings	Maternal PTSD and depression	↑ emotional reactivity and aggression (41)
Terrorism (277 Israeli adolescents)	Self-report, checklist	Maternal warmth and authoritativeness buffer; authoritarianism worsens	Authoritative parenting→ ↓ symptoms; authoritarian parenting → ↑ externalizing behaviors (43)
Loss of a loved one			
Bereaved caregivers (74 adults and children)	Caregiver self-report	↑ caregiver self-compassion → ↓ grief/distress, ↑ warmth and consistency	↓ internalizing and externalizing symptoms (47)
Acute medical injury			
Severe maternal illness (35 women and 11 partners)	Interviews	ICU stay and medical instability, disrupted caregiving; separation limited bonding	Delayed attachment, breastfeeding difficulties, and inconsistent caregiving (53)

↑, increase; ↓, decrease; PTSD, posttraumatic stress disorder; TBI, traumatic brain injury.

This table summarizes studies examining how acute trauma exposures influence parenting behaviors and child outcomes. For each study, the development window of exposure or assessment (prenatal, early childhood, school age) is indicated, as timing plays an important role in shaping both caregiving responses and offspring vulnerability. Across studies, trauma-related distress is linked to heightened parental anxiety, inconsistent caregiving, and reduced warmth, contributing to child emotional reactivity, behavioral problems, and attachment disruptions. Parenting styles moderate these effects: authoritative (warm, structured) parenting is associated with fewer symptoms, whereas authoritarian (high control, low warmth) parenting predicts greater externalizing behaviors. These studies were included to contextualize trauma-related caregiving behaviors and offspring outcomes within psychosocial pathways interacting with biological and epigenetic processes.

## 3 Chronic trauma

Across different forms of chronic trauma, prolonged or repeated stress exposure has been associated with cumulative biological and psychological adaptation that develops over time. Evidence from multiple trauma contexts points to involvement of interacting regulatory systems, including stress-response signaling, neurodevelopmental processes, metabolic regulation, and immune-inflammatory activity (54–81). The duration and timing of exposure, as well as cumulative stress burden, appear to influence how these biological responses emerge, while environmental conditions and individual adaptation contribute to variability in outcomes. Caregiving and social environments also play an important role, as chronic hardship may disrupt emotional availability or promote adaptive regulation and resilience.

### 3.1 Domestic violence or intimate partner violence

Domestic violence and intimate partner violence (IPV) represent chronic interpersonal stressors associated with sustained psychological and physiological dysregulation. Evidence across IPV-related exposures implicates interacting biological systems, particularly stress-response regulation, neurodevelopmental signaling, metabolic processes, and immune-inflammatory activity (54–57). Repeated exposure to threatening or coercive environments may promote cumulative epigenetic regulation consistent with chronic stress physiology and allostatic load.

Chronic IPV exposure has been associated with epigenetic modification of stress-regulatory and neural plasticity pathways. Multigenerational findings demonstrate differential DNA methylation in genes involved in synaptic signaling and mitochondrial regulation, including *BDNF* and *CLPX*, supporting coordinated involvement of neurodevelopmental and metabolic systems (54). Additional studies link IPV exposure to altered methylation of *NR3C1*, corresponding with differences in stress reactivity and anxiety-related symptoms (55). These molecular patterns are not uniformly observed across generations, suggesting chronic interpersonal stress may shape biological vulnerability through gradual, context-dependent regulatory processes.

Psychosocial and caregiving systems appear closely integrated with these biological responses. Persistent IPV exposure has been associated with elevated maternal psychological distress, including depressive and anxiety symptoms, corresponding with disruptions in emotional regulation and increased behavioral difficulties in children (56). Longitudinal evidence further demonstrates reduced parental sensitivity, warmth, and responsiveness, caregiving patterns linked to variation in children’s executive functioning and emotional development (57).

IPV models suggest prolonged interpersonal threat may gradually change stress-regulatory and neurodevelopmental systems, while metabolic and caregiving pathways influence whether these adaptations contribute to vulnerability or resilience.

### 3.2 Living in a war zone or refugee camp long-term

Prolonged exposure to war and displacement reflects chronic environmental stress characterized by sustained physiological adaptation. Current evidence implicates stress-response regulation, neurodevelopmental signaling, metabolic regulation, and inflammatory activation (58–62). Recurrent exposure to instability and threat appears to promote progressive biological embedding through cumulative epigenetic regulation, consistent with chronic stress physiology.

Across war-exposed populations, chronic trauma has been associated with epigenetic modulation of pathways governing cellular metabolism, neural development, and adaptive stress responses. Differential DNA methylation in genes involved in mitochondrial energy regulation, intracellular transport, and neurodevelopment suggests integration of metabolic and neuroregulatory systems under prolonged adversity (58, 59). These molecular patterns vary by trauma type, exposure timing, and biological sex, indicating context-dependent regulatory responses rather than uniform biological effects (59). Alterations in epigenetic aging, including both accelerated and delayed biological aging, further suggest disruption of developmental timing and system-level physiological regulation under sustained stress (58, 59).

Multigenerational findings demonstrate shared involvement of stress-regulatory and inflammatory systems across exposure types. Consistent directional methylation changes across direct, prenatal, and germline exposure, together with dose-response relationships between trauma burden and molecular changes, support cumulative biological adaptation to prolonged adversity (59). However, variability across populations highlights the influence of environmental and methodological factors (60).

Psychosocial and caregiving systems remain closely integrated with these biological processes. Chronic displacement is associated with caregiver psychological distress, emotional dysregulation, and reduced caregiving stability (61, 62). These caregiving changes correspond with increased emotional and behavioral vulnerability in children, even in the absence of direct trauma exposure (61, 62). Caregiver regulation and environmental stability appear to moderate developmental outcomes under sustained adversity.

War and displacement models suggest sustained environmental threat may shift stress-regulatory and metabolic set points over time. Cumulative exposure burden and caregiving stability appear to shape long-term regulation.

### 3.3 Long-term neglect or abandonment

Chronic caregiving deprivation represents sustained developmental stress associated with long-term alteration in emotional, behavioral, and physiological regulation. Neglect-related exposures point to involvement of stress-regulatory and neurodevelopmental systems, reflecting adaptive responses to prolonged early-life adversity (63–68). Disruption of caregiving during sensitive developmental windows may shape enduring regulatory patterns.

Experimental models of early-life neglect demonstrate coordinated changes in stress-response and neurodevelopmental signaling following repeated maternal separation and reduced caregiving. These models show heightened stress reactivity, impaired emotional regulation, and persistent behavioral differences associated with altered expression of stress-related genes (63). Multigenerational early-life stress models demonstrate enduring regulatory and behavioral variation, including depressive-like behaviors and altered responses to environmental novelty (64). These findings include epigenetic modulation of genes involved in synaptic and stress signaling, including *Mecp2*, *CB1*, and *CRFR2*, with methylation differences observed in both germline and neural tissues (64). Persistence of these patterns across generations, even without continued stress exposure, suggests stable regulatory shifts rather than transient activation.

Human studies demonstrate similar involvement of stress-regulatory neuroendocrine systems. Childhood neglect has been associated with increased methylation of *NR3C1*, corresponding with altered glucocorticoid signaling and reduced flexibility of HPA axis regulation (65). Coordinated epigenetic variation across multiple stress-regulatory genes, including *FKBP5* and *CRHR1*, supports system-level modulation of stress-response pathways, although variability across generations suggests transmission may depend on environmental context (66).

Chronic neglect also influences psychosocial and caregiving regulation. Individuals with histories of early neglect are more likely to demonstrate reduced emotional engagement, diminished responsiveness, and difficulty interpreting children’s emotional cues, caregiving patterns linked to differences in offspring emotional and behavioral regulation (67). These vulnerabilities may be amplified by co-occurring psychological distress and environmental strain, whereas emotionally supportive caregiving appears to buffer intergenerational risk (68).

Neglect models suggest sustained caregiving deprivation during sensitive developmental windows may relate to changes in stress-regulatory and neurodevelopmental systems. Intergenerational outcomes may be influenced by caregiving stability.

### 3.4 Persistent food insecurity or starvation

Chronic nutritional deprivation represents sustained metabolic stress associated with long-term disruption of physiological regulation. Evidence across famine-related exposure suggests coordinated involvement of metabolic programming, stress-response regulation, neurodevelopmental processes, and inflammatory signaling (69–76). When occurring during pregnancy, altered energy availability and endocrine signaling may influence fetal developmental programming.

In famine-exposed populations, chronic undernutrition has been associated with alterations in neurodevelopmental and stress-regulatory systems. Prenatal famine exposure corresponds with long-term variation in cognitive and emotional regulation consistent with altered neural and glucocorticoid signaling pathways (69, 70). Multigenerational findings demonstrate variation in growth, renal function, and mortality, suggesting persistent metabolic and physiological adaptation following early nutritional deprivation (71–74). These findings are consistent with developmental programming influenced by exposure, severity, timing, and environment.

Chronic nutritional stress also interacts with psychosocial and caregiving systems. Histories of food insecurity are associated with elevated parental distress and caregiving strain, corresponding with variation in emotional responsiveness and caregiving stability (75). Parents exposed to early undernutrition may also demonstrate altered feeding-related regulation behaviors, suggesting interaction between metabolic stress history and caregiving patterns (76).

Famine and food insecurity models suggest prolonged nutritional deprivation may relate to alterations in metabolic, stress-regulatory, neurodevelopmental, and psychosocial systems.

### 3.5 Housing instability or homelessness

Chronic housing instability reflects sustained environmental stress associated with prolonged activation of stress-responsive physiological systems. Evidence suggests involvement of stress-response regulation, inflammatory signaling, metabolic processes, and neuroendocrine pathways, consistent with cumulative stress physiology (77–81).

Housing instability has been associated with dysregulation of neuroendocrine and inflammatory systems. Accelerated epigenetic aging suggests prolonged activation of stress and immune pathways, reflecting cumulative physiological burden (77). Associations between poor housing quality, depressive symptoms, and epigenetic variation further support interaction between chronic psychosocial stress, inflammatory signaling, and molecular stress regulation (78).

During pregnancy, housing instability may influence fetal developmental programming through stress-mediated physiological pathways. Maternal exposure to eviction has been associated with altered fetal growth and shortened gestation, suggesting disruption of placental, metabolic, and endocrine regulation (79).

Psychosocial and caregiving systems interact with these biological processes. Chronic housing instability is associated with elevated caregiver stress, altered emotional regulation, and competing survival demands, which may influence caregiving consistency and responsiveness (80). Disrupted routines and environmental unpredictability may further affect early neurobehavioral regulation and stress-response development in children (81).

Housing instability models suggest sustained environmental unpredictability may maintain prolonged stress-response and inflammatory activation. The timing of exposure and caregiving environment appear to influence how these interacting regulatory processes contribute to long-term development.

When considered as a whole, chronic trauma models suggest prolonged stress exposure engages multiple regulatory systems over time, with developmental outcomes shaped by cumulative burden, exposure timing, and caregiving context (**Tables 3**, **4**).

**Table 3:** Epigenetic Mechanisms Linking Chronic Trauma to Offspring Development.

Exposure/population	Sample type	Epigenetic changes	Consequences
Domestic violence or intimate partner violence			
Domestic violence (375 participants, 3 gen)	Saliva	Methylation changes at *BDNF* and *CLPX*	Altered neural development and stress regulation (54)
IPV(20 mother child pairs vs. controls)	Saliva	↑ *NR3C1* methylation (mothers)	↑ Maternal anxiety, no significant child effect (55)
Living in a war zone or refugee camp long-term			
Refugee (1,507 Syrian children in Lebanon)	Saliva	Sex-specific methyl change in neurological-related genes	Epigenetic age deceleration (58)
Refugee (48 Syrian families, 3 gen)	Buccal swabs	35 DMR across generations	Epigenetic age acceleration (prenatal) (59)
Refugee (207 Burundian caregivers and children)	Buccal swabs	No significant methylation change	No significant changes noted (60)
Long-term neglect or abandonment			
Adults w/childhood neglect (215 adults)	Blood	↑ *NR3C1* exon 1F methylation	HPA axis dysregulation (65)
Mother w/childhood neglect (113 mother-infant pairs)	Maternal blood, cord blood	↑ *NR3C1* methylation, ↓ *FKBP5* and ↓ *CRHR1* methylation (mothers); no infant change	Maternal HPA axis dysregulation, unaffected infant (66)
Persistent food insecurity or starvation			
Famine Exposure (5,150 various exposure participants)	Survey data, cognitive testing	Altered methylation in stress/cognition pathways	↑ risk of depression, ↓ cognition (69)
Famine Exposure (923 before conception vs. pregnancy)	Self-reports, blood	Altered mood/stress genes	↓ mental health, ↑ depression, ↓ quality of life (70)
Famine Exposure (3,734 various exposure participants)	Birth years, BMI	Altered nutrition genes	Altered BMI trajectories (71)
Famine Exposure (3 generations)	Harvest/mortality records	Altered metabolic/stress genes	↑ cancer and mortality in grandsons (72)
Famine Exposure (31,449 women)	Birth records	Altered growth pathways	↓ birth size exposed offspring (73)
Siege of Leningrad survivors (offspring vs. control)	Clinical Data	Altered dietary patterns	↓GFR, ↑ creatinine → long term renal dysfunction (74)
Housing instability or homelessness			
Housing instability (1,420 adults)	Blood	Accelerated DNA methylation aging	↑ risk poor health outcome (77)
Evicted mothers (88,862 infants)	Birth Records	Stress pathway disruption	↓BW, ↓ gestation, ↑ premature risk (79)

↑, increase; ↓, decrease; NM, not measured; BW, birth weight; CpG, cytosine-phosphate-guanine site; DMR, differentially methylated region; GFR, glomerular filtration rate; BMI, body mass index.

This table summarizes studies examining how chronic trauma exposures influence offspring development through epigenetic pathways. Across studies, prolonged stressors are linked to DNA methylation changes, altered gene expression, and accelerated epigenetic aging. These biological disruptions contribute to dysregulated stress and immune responses, cognitive and emotional difficulties, and heightened risk for long-term health problems. Select studies without direct epigenetic measurements were included when offspring outcomes have been independently linked to trauma-associated epigenetic mechanisms in related populations.

**Table 4:** Parenting and offspring consequences of chronic trauma.

Exposure/ population	Method	Parenting factors	Child outcomes
Domestic violence or intimate partner violence			
IPV Exposure (154 families)	Questionnaires, observations	↓ maternal warmth, attentiveness, and responsiveness	↓ executive functioning at school entry (57)
Living in a war zone or refugee camp long-term			
Refugees (291 Syrian mothers in Lebanon)	Structured interviews, SEM modeling	↑maternal distress, harsher/rejecting parenting	Psychosocial difficulties and poor adjustment (61)
Long-term neglect or abandonment			
Neglect Exposure (138 exposed vs. control)	Questionnaires, observed interactions	↓ involvement and ↓ responsiveness	Weak bonding, ↑ developmental risks (67)
Neglect Exposure (140 exposed vs. control)	Healthcare/home observations	Disengaged and emotionally unavailable parenting	↑ risk of neglect cycle (69)
Persistent food insecurity or starvation			
Food insecurity (2,870 mothers)	Surveys	↑ maternal depression/anxiety, ↓ warmth, inconsistency	↑ aggression, anxiety, depression, and inattention (75)
Food insecurity (702 WIC mothers)	Telephone survey	↑ maternal stress → restrictive feeding practices	Feeding problems, ↓ autonomy, and ↓ emotional security (76)
Housing instability or homelessness			
Housing instability (200 children)	Observations, questionnaires	↓ consistent and ↓ warm caregiving	↑ reactivity, ↓ adaptability, ↓ security (80)
Housing instability (59 low-income mothers)	Observations	Homeless mothers → ↓ stimulation, ↓ warmth,	Emotional regulation difficulties, trust issues (81)

↑, increase; ↓, decrease.

This table summarizes studies on how chronic trauma influences parenting behaviors and offspring outcomes. Across studies, prolonged adversity is linked to heightened parental stress, reduced warmth and consistency, and emotionally unavailable caregiving. These patterns contribute to greater child emotional reactivity, difficulties with trust and regulation, and greater psychosocial risk. These studies were included to contextualize trauma-related caregiving behaviors and offspring outcomes within psychosocial pathways interacting with biological and epigenetic processes.

## 4 Complex trauma

Across diverse forms of complex trauma, sustained exposure to interpersonal and collective adversity has been associated with cumulative biological and psychological adaptation that may extend across generations. Evidence from multiple trauma contexts points to involvement of interacting regulatory systems, including stress-response signaling, neurodevelopmental processes, circadian and metabolic regulation, and epigenetic remodeling (7, 82–124). The severity, developmental timing, and cumulative burden of exposure appear to influence how these biological responses emerge over time, with sociocultural and environmental stability contributing to variability in outcomes.

### 4.1 Genocide

Genocide represents one of the most severe forms of sustained trauma, involving persecution, displacement, and prolonged threat to survival. Across survivor populations and their descendants, research suggests coordinated involvement of stress-response regulation, neurodevelopmental signaling, and behavioral adaptation (7, 82–100). Exposure severity, developmental timing, and cumulative stress burden appear to influence how these biological responses emerge across generations.

Alterations in stress-response regulation are among the most consistently reported findings in genocide-related research. Studies of Holocaust survivors and their offspring demonstrate differential DNA methylation of key HPA-axis genes, including *NR3C1* and *FKBP5*, corresponding with variation in glucocorticoid receptor sensitivity and cortisol feedback regulation (82, 83). Divergent patterns have been observed depending on parental PTSD status, with paternal PTSD in the absence of maternal PTSD associated with increased methylation of *NR3C1* in offspring, whereas combined maternal and paternal PTSD has been associated with decreased methylation (82). Variation in *FKBP5* methylation across intronic regions has also been reported, with opposite directional patterns observed in survivors and their offspring, corresponding with differences in *FKBP5* expression and glucocorticoid receptor sensitivity (83, 84). Functional alterations in cortisol metabolism, including reduced cortisol excretion in survivors and increased activity of cortisol-inactivating enzymes in offspring, have also been described (82, 85). Similar involvement of stress-response pathways has been observed in survivors of the Tutsi genocide and related conflicts, where methylation differences in *NR3C1* and *NR3C2* have been reported (88, 89).

Genocide exposure has also been associated with epigenetic variation in pathways involved in neurodevelopment and memory processing. Methylation differences in survivors of the Tutsi genocide and related conflicts have been reported in genes regulating synaptic plasticity, neurotrophic signaling, and early developmental programming (88–92). Variation at the NGFI-A binding site of *NR3C1* has been associated with altered memory processing and sex-specific differences in PTSD risk, with reduced intrusive recall observed in males and lower PTSD risk reported in females (90, 91). Methylation differences in *NTRK2*, a gene involved in synaptic signaling and memory formation, have been linked to variation in recognition memory performance and lifetime PTSD risk (92). Genome-scale analyses have further identified methylation changes in genes involved in embryonic and neural development, including *BCOR*, *PRDM8*, and *VWDE*, in offspring with *in utero* exposure to the Tutsi genocide (7).

In addition, behavioral and metabolic adaptations have been described in descendants of famine-associated genocides such as the Holodomor. Second and third generation descendants have reported persistent “survivor mode” behaviors characterized by fear, hypervigilance, food hoarding, and overeating, despite not directly experiencing the original trauma (93). Specific epigenetic mechanisms were not identified in these reports, although the persistence of these behavioral patterns across generations may reflect stress-response and metabolic adaptation (93).

Caregiving regulation represents an additional pathway through which genocide-related trauma may influence intergenerational outcomes. Across genocide-affected populations, parental exposure has been associated with altered caregiving patterns characterized by overprotection, controlling behaviors, diminished warmth, and role-reversal dynamics (94–97, 100). Maternal survivor status has been more strongly associated with offspring psychological vulnerability compared to paternal exposure, with dual parental exposure corresponding to the highest reported risk (94). These caregiving patterns have been linked to increased internalizing and externalizing symptoms, ambivalent attachment styles and altered self-perception in children and grandchildren (95, 96). In some contexts, including families affected by the Khmer Rouge genocide and Rwandan genocide, parental PTSD and maternal violence have been associated with heightened anxiety, depression, and antisocial behaviors in offspring (97–99). Increased medical and psychiatric medication use observed among offspring of Holocaust survivors further suggests broader psychosocial and health-related vulnerability (100).

Genocide models suggest sustained exposure to extreme threat may be associated with coordinated stress-response and neurodevelopmental regulation across generations, with intergenerational outcomes shaped by parental psychological status and caregiving stability.

### 4.2 War violence and trauma related to indigenous communities

War violence and historical trauma involve sustained exposure to armed conflict, forced displacement, and collective sociopolitical disruption across generations. These exposures have been associated with coordinated alterations in stress-response regulation, circadian and sleep-related pathways, and metabolic processes (102–115). The timing, duration, and cumulative burden of exposure appear to influence how these biological changes develop over time.

Alterations in stress-response regulation appear to be among the most consistent findings in war-exposed populations and indigenous communities affected by historical trauma. Offspring of veterans with PTSD have demonstrated lower cortisol levels compared to controls, while epinephrine and norepinephrine levels were unchanged (102). These differences have been associated with methylation changes in *NR3C1*, a key regulator of HPA-axis feedback (103). Similarly, maternal exposure to Canadian residential schools has been linked to higher cortisol, catecholamines, and inflammatory cytokine levels in offspring, including cases in which offspring were not raised by their biological parents (104, 105). Elevated adverse childhood experience scores and poorer mental and physical health outcomes have also been documented in second and third generation descendants of residential school survivors (106). Population-level studies further suggest intergenerational stress-related effects of war trauma, including higher psychiatric hospitalization rates among daughters of evacuated Finnish mothers and increased mortality among sons of former prisoners of war (107, 108).

Circadian and sleep-related pathways have also been implicated in war-related trauma. Differential methylation of sleep and circadian genes such as *PAX8* and *LHX1* has been reported in veterans with PTSD (103, 109–111). These changes have been associated with variation in memory processing, sleep duration, and circadian rhythm regulation, as well as differences in PTSD symptom severity (103, 109–111). Disruption of circadian regulation may represent a pathway of stress-related vulnerability following trauma exposure; however, evidence in offspring remains limited.

Metabolic and broader regulatory pathways have also been implicated particularly in studies of indigenous communities experiencing historical trauma (112). Genome-wide analyses in Alaska Native communities have identified methylation differences in genes involved in mitochondrial metabolism, calcium transport, chromatin organization, and molecular trafficking (8). These findings implicate coordinated variation across metabolic and regulatory pathways and have been associated with historical loss-related symptom reporting (8).

Caregiving environments represent an additional pathway through which war-related trauma may influence intergenerational outcomes. Across conflict-affected populations, exposure to active combat or chronic threat has been associated with harsh, inconsistent, diminished warmth, or overprotective parenting patterns (113–115). These caregiving styles have corresponded with increased internalizing and externalizing symptoms in offspring, reduced parental bonding, and family dysfunction (114, 115). Fluctuating parenting behaviors characterized by shifts between warmth, avoidance, and control have also been reported in families exposed to ongoing conflict (114).

War violence and historical trauma models suggest sustained exposure to conflict and sociocultural disruption may be associated with coordinated changes in stress-response, circadian, and metabolic systems across generations.

### 4.3 Childhood maltreatment

Childhood maltreatment, including abuse and neglect, represents a sustained early-life stressor associated with long-term biological and behavioral changes across generations. Evidence suggests involvement of stress-response regulation, neurodevelopmental and emotional processing pathways, and epigenetic remodeling processes in shaping intergenerational outcomes (116–124). Exposure timing, severity, and duration appear to influence how these biological responses are expressed over time.

Experimental models have provided mechanistic insight into how early-life stress may influence intergenerational outcomes. In animal studies using the maternal separation with unpredictable stress (MSUS) paradigm, exposed males demonstrated alterations in sperm small and long noncoding RNAs (116, 117). Similar RNA changes were detected in offspring tissues, including brain and serum (116). Behavioral and metabolic phenotypes were observed in subsequent generations, and injection of sperm RNA from exposed males into naive zygotes reproduced several of these features (116, 117). Animal models have also demonstrated epigenetic remodeling of stress-related genes and histone acetylation patterns in brain regions involved in emotional regulation following early-life stress (116, 117).

Human studies have identified epigenetic variation in stress-response regulatory systems among individuals exposed to childhood maltreatment. Altered methylation of genes involved in HPA-axis feedback and glucocorticoid homeostasis, including *NR3C1* and *FKBP5*, has been reported in individuals with histories of abuse or neglect (118–120). Lower methylation of *FKBP5* has been associated with structural variation in brain regions involved in emotional regulation (120, 121). Increased methylation of the *NR3C1* promoter and reduced glucocorticoid receptor expression have been observed in hippocampal tissue of suicide completers with histories of maltreatment (119, 120). Epigenetic variation in glucocorticoid regulatory pathways has been described in this context; however, evidence directly linking these changes to intergenerational suicide risk remains limited.

Psychosocial and caregiving systems remain closely integrated with these biological processes. Individuals with histories of maltreatment have demonstrated reduced parental competence, diminished emotional support, and increased harsh or neglectful parenting behaviors (122–124). Paternal maltreatment history has been associated with increased externalizing behaviors in offspring, partially mediated by harsh parenting (123). Elevated personal distress and depressive symptoms in maltreated parents have also been associated with dysfunctional caregiving patterns (124). Variability in caregiving stability and emotional regulation may interact with stress-related biological vulnerability in shaping psychological outcomes in children.

Childhood maltreatment models suggest early-life adversity may be associated with coordinated alterations in stress-response regulation, neurodevelopmental signaling, epigenetic regulation, and caregiving environments across generations.

When considered collectively, complex trauma models indicate that sustained interpersonal and sociopolitical adversity may involve coordinated regulatory changes across generations, with intergenerational patterns shaped by cumulative burden, developmental timing, and caregiving stability (**Tables 5**, **6**).

**Table 5:** Epigenetic Mechanisms Linking Complex Trauma to Offspring Development.

Exposure/population	Sample type	Epigenetic changes	Consequences
Genocide			
Holocaust (80 prenatally exposed offspring vs. 15 controls)	Blood	Altered methylation *GR-1F promoter of NR3C1*	↑ PTSD risk (mediated by paternal PTSD) or *↓* PTSD risk (mediated by maternal PTSD) (82)
Holocaust (32 survivors and 22 prenatally exposed offspring)	Blood, Saliva	↑ *FKBP5* methylation in survivors, *↓ FKBP5* methylation in offspring	↑ PTSD risk in survivors, ↓ PTSD risk in offspring (83)
Holocaust (85 prenatally exposed offspring)	Urine	↑11β-HSD-2 activity	*↓* stress sensitivity (86)
Tutsi Genocide (25 pregnant women and prenatally exposed offspring)	Blood	↑ methylation *NR3C1 exon 1* , ↑ *NR3C2* methylation	↓ stress-related conditions, ↓ PTSD (88)
Tutsi Genocide (152 middle-aged survivors)	Saliva, fMRI	↑ methylation at the *NGFI-A* binding site of the *NR3C1 promoter*	↓ PTSD risk in females; impaired memory in males (90)
Tutsi Genocide (20 pregnant women and 16 prenatally exposed offspring)	Blood	↑ methylation of *BCOR, PRDM8, VWDE*	Altered embryonic/neural development, ↑ depression risk (7)
Tutsi and Ugandan Genocide (350 Tutsi and 463 Ugandans across all age groups)	Saliva	↑ methylation of *NTRK2*	Impaired memory formation, ↑ PTSD risk (9)
War violence and trauma related to indigenous communities			
117 Alaskan Natives (all adults across different age groups)	Blood	↑ methylation at *SGK1, MAPK10, CREBBP, HSP90AA1,DENND1A, ATP2B4, PCBP3*	Altered chromatin organization, stress response, cellular metabolism (8)
Childhood maltreatment			
MDD and maltreatment (60 adults aged between 18 and 65)	Blood, fMRI	↓ methylation of *FKBP5 intron 7*	↑ depression risk, altered brain structure (119)
Childhood abuse (12 adult suicide victims)	Hippocampus tissue (Postmortem)	↑ methylation of the *NR3C1 promoter*	↑ suicide risk (120)

↑, increased; ↓, decreased; NM, Not Measured; PTSD, Post-Traumatic Stress Disorder.

Complex transgenerational trauma has profound impacts on genes involved in the HPA axis, memory function, embryonic development, and other biological pathways. These changes have been linked to increased susceptibility to various psychiatric disorders, such as PTSD, depression, and the risk of suicide. Select studies without direct epigenetic measurements were included when offspring outcomes have been independently linked to trauma-associated epigenetic mechanisms in related populations.

**Table 6:** Parenting and offspring consequences of complex trauma.

Exposure/ population	Method	Parenting factors	Child outcomes
Genocide			
Holocaust (137 survivors aged 23 to 65)	Observations	NM	↑ use of psychotropic, antihypertensive, and lipid-lowering medications (100)
Holocaust (599 prenatally exposed children and 311 grandchildren)	Observations, questionnaire	↑ controlling or overprotective parenting	↑ anxiety, ↑ depression, ↑ PTSD (95)
Holocaust (88 middle-class families, 2 generations prenatally exposed)	Observations	↓ acceptance and encouragement of independence	↑ ambivalent attachment styles (96)
Cambodian Genocide (46 prenatally exposed female high school students and their survivor mothers)	Observations	Maternal PTSD and role-reversal parenting	↑ anxiety in daughters (97)
Rwandan Genocide (125 survivor mothers and their 12 -year-old children born 2 years after the genocide)	Observations, interview	Maternal PTSD, family violence	↑ depression, anxiety, and antisocial behaviors (98)
Holodomor genocide (15 Ukrainian families, prenatally exposed adult child and grandchild)	Observations, interview	Constellation of emotions, inner states, and trauma coping strategies	Live in “survivor mode”, food hoarding, overeating (93)
War violence and trauma related to indigenous communities			
Beirut clashes (28 parents, 24 postnatally exposed adolescent offspring)	Observations, interview	Parenting styles of warmth, avoidance, and control	NM (114)
Croatian War (122 war veterans with PTSD and their prenatally exposed adolescent offspring)	Observations, questionnaire	Overprotective, overcontrolling	↑ internalizing and externalizing, ↓ parental bonds (115)
Childhood maltreatment			
Childhood maltreatment (489 parents of children aged 5 to 13)	Observations, questionnaires	↓ parental competence	↓ effective communication and social support (122)
Childhood maltreatment (13 mothers vs 42 controls, aged 25 to 50)	Observations, questionnaires	↑ personal distress	↑ risk of depression (124)

↑, increased; ↓, decreased; NM, Not measured; PTSD, Post-Traumatic Stress Disorder.

Complex transgenerational trauma markedly alters parenting styles, manifested as parental PTSD, family violence, increased parental stress, increased affective empathy, decreased parental competence, overprotective, overcontrolling, and role-reversing parenting. Direct consequences of these dysfunctional parenting styles away from the norm in their offspring include, but are not limited to, increased risk of depression, PTSD, and anxiety, and decreased bond between parent and child. These studies were included to contextualize trauma-related caregiving behaviors and offspring outcomes within psychosocial pathways interacting with biological and epigenetic processes.

## 5 Treatment approach for multi-generational trauma

Effective interventions for multi-generational trauma often require addressing both individual psychiatric symptoms and the family environments through which trauma-related vulnerability is expressed and reinforced (125). Prevention and treatment approaches therefore tend to span multiple levels, including trauma-focused therapy, attachment and relationship-based interventions, and broader strategies that support regulation through sleep, stress management, and health behaviors (19, 125–135). As trauma biology becomes better characterized, there is also growing interest in whether trauma-associated epigenetic profiles could help refine treatment selection or predict response (136, 137). In this section, prevention strategies, current psychotherapy approaches, and emerging biologically informed treatment directions relevant to intergenerational trauma-related risk are discussed.

Prevention remains a central strategy for reducing intergenerational risk, particularly when it targets unresolved trauma symptoms and early relational functioning (19). Two commonly described prevention targets include trauma-specific interventions in adults and attachment-focused interventions within families (19). Trauma-focused care for adults with severe and persistent trauma-related distress, including chronic childhood maltreatment, may reduce symptom burden that interferes with parenting capacity (126). Attachment-based approaches that strengthen caregiver attentiveness and reflective functioning during the postpartum period have been associated with improved attachment outcomes in high-risk families, which may reduce downstream developmental vulnerability (127). These strategies align with the broader framework that early intervention on caregiver distress and relational functioning may reduce the persistence of trauma-related risk across generations (19).

Although preventative interventions demonstrate clinical benefit, no studies directly evaluate whether these approaches modify trauma-associated epigenetic variation at specific gene targets. However, lifestyle and behavioral factors known to influence epigenetic regulation, including diet, sleep, stress management, and substance use, may represent modifiable contributors to biological vulnerability (128). Across multiple trauma contexts, altered methylation of *NR3C1* has been reported, including genocide exposure, childhood maltreatment, and natural disasters (10, 82, 88, 89). *NR3C1* also appears responsive to environmental inputs, suggesting that trauma-associated epigenetic patterns may remain dynamically regulated rather than fixed. In this context, dietary patterns may also play a role, including the consumption of industrialized foods such as sausages, sugary drinks, and chocolate-based products, which have been associated with increased *NR3C1* methylation (129). These findings support consideration of health behavior interventions as adjunctive strategies for modulating stress-response regulatory pathways to help mitigate potential trauma-associated epigenetic vulnerability linked to *NR3C1*.

The current literature on intervention strategies has been most developed in the context of childhood maltreatment and disrupted caregiver-child relationships. Several approaches emphasize caregiver regulation and family functioning in addition to symptom reduction. Multi-family therapy models focused on emotional regulation, mentalization, and empowerment have been associated with improved parent-child relationship functioning and reduced trauma-related vulnerability (125). Child-parent psychotherapy, which addresses maladaptive trauma-related beliefs and promotes relational safety, has shown benefit for young children and caregivers (130). The Mom Power program, integrating clinician-guided self-care, social support, and parenting skills, has been associated with reductions in maternal depression and PTSD symptoms and improvements in attachment-related outcomes (131). These findings indicate that structured, relationship-centered programs can improve caregiving functioning and child emotional outcomes in families affected by intergenerational trauma (125, 130, 131).

Interventions for trauma related to war and displacement similarly focus on reducing child PTSD symptoms while restoring environmental predictability and safety (132). Cognitive behavioral therapy remains a core evidence-based treatment in this context, often supported by stable school and community environments (133). Parental communication that contextualizes war experiences has been associated with lower psychiatric stress and PTSD symptoms in offspring (134). Alternative approaches, including role play, drama, and art-based therapy, have also demonstrated potential benefits (135). Whether these symptom-focused improvements correspond with measurable changes in biological embedding, including epigenetic variation, remains unclear and represents an important direction for future research.

Emerging evidence suggests trauma-associated epigenetic variation may influence treatment responsiveness rather than serve as a direct therapeutic target. Decreased methylation of *FKBP5* intron 7 has been associated with improved response to exposure-based cognitive behavioral therapy in anxiety disorders (136). *FKBP5* methylation differences have been reported across multiple trauma contexts in both survivors and offspring, raising the possibility that stress-response regulatory genes may help distinguish individuals more likely to benefit from specific interventions (38, 66, 83, 84, 136). Narrative exposure therapy has been associated with increased *NR3C1* methylation in treatment responders but not in non-responders among war survivors (137). Some findings do not correspond directly with gene expression differences, suggesting that mechanisms beyond simple transcriptional regulation may contribute to treatment response (136). Integration of epigenetic profiling with established psychotherapies may help refine treatment stratification in the future, although current evidence remains preliminary.

## 6 Limitations and controversies

Interpretation of multi-generational trauma research requires careful consideration of several methodological and conceptual limitations. Recurring concerns include candidate gene bias, sampling constraints, tissue specificity, temporal variability in epigenetic measurement, and the biological implication of post-fertilization epigenetic reprogramming. These factors complicate efforts to determine the stability, specificity, and functional relevance of reported epigenetic associations.

One of the most persistent methodological concerns is candidate gene bias. A substantial proportion of studies have focused on a limited number of stress-response genes, most notably *FKBP5* and *NR3C1* (55, 65, 83, 84, 121, 129). Many of these investigations examine specific loci within these genes, such as discrete intronic regions, rather than adopting an epigenome-wide approach. While hypothesis-driven candidate gene studies can provide mechanistic insight, narrow locus selection risks overemphasizing the importance of specific genes while overlooking broader regulatory networks (83, 84, 121, 129). Epigenome-wide analyses are therefore needed to reduce selection bias and more comprehensively characterize trauma-associated regulatory variation across the genome.

Participant sampling presents unique challenges for the evaluation of the transmission of trauma. Intergenerational humans are more common than true transgenerational studies, which often rely on animal models due to practical constraints in recruiting participants across multiple generations. Human sample sizes are frequently modest, with many studies enrolling fewer than 100 participants (38, 81, 114). Limited sample size reduces statistical power and constrains generalizability, particularly in the context of epigenetic variation, which is often subtle and influenced by numerous environmental and biological covariates. Larger, multi-site cohorts will be essential for validating reported associations and clarifying effect sizes.

Population selection further complicates interpretation. Many investigations focus on historically defined trauma-exposed populations, including Holocaust survivors, Rwandan genocide survivors, and Alaska Native communities (8, 82, 98). Although these cohorts are critical for understanding trauma-related outcomes, shared ancestry and sociocultural context may introduce confounding variables. Distinguishing trauma-associated epigenetic variation from population-level background variation remains challenging. Inclusion of carefully matched control groups and replication across diverse populations are necessary to strengthen causal inference.

Tissue specificity and timing of sample collection introduce additional variability. Epigenetic regulation is highly tissue-dependent, and most human studies rely on accessible peripheral tissues such as blood, saliva, or urine (138). Methylation patterns in these tissues may not fully reflect epigenetic processes occurring in the brain or germline tissues. Moreover, epigenetic marks are dynamic and may evolve over time following trauma exposure. Differences in timing of sample collection, whether shortly after exposure or years later, can influence reported associations, making cross-study comparisons difficult. Standardization of sampling protocols and longitudinal designs would improve interpretability.

Contextual heterogeneity across trauma types also limits generalization. Even when examining the same gene, findings may differ depending on the nature of the traumatic exposure. For example, increased methylation of *NR3C1* has been reported in both earthquake survivors and survivors of the Tutsi genocide, yet associations with cognitive and psychiatric outcomes differ across contexts (10, 88). These differences highlight the importance of exposure characteristics, developmental timing, and co-occurring environmental factors in shaping downstream biological and behavioral outcomes. Interpretation of locus-specific methylation differences should therefore remain context-dependent rather than generalized across trauma types.

Beyond methodological concerns, conceptual controversy persists regarding the feasibility of transgenerational epigenetic inheritance in humans. Post-fertilization epigenetic reprogramming involves widespread DNA demethylation of paternal and maternal genomes, followed by remethylation during early embryogenesis (5). A second wave of demethylation occurs during primordial germ cell development, including erasure of many parental imprints (5). These processes raise questions about whether environmentally induced methylation changes can persist across generations. However, accumulating evidence suggests that epigenetic reprogramming is not absolute. Certain genomic regions, including imprinted loci, non-imprinted loci, and retrotransposable elements, may partially escape complete demethylation (139, 140). In addition, experimental studies demonstrate that sperm-derived small noncoding RNAs, including miRNAs, tsRNAs, and lncRNA, can transmit information about paternal stress exposure independently of stable DNA methylation changes (139, 140). These RNA-mediated pathways influence neurodevelopmental, metabolic, and stress-related phenotypes in offspring in animal models. Such findings suggest that germline transmission of trauma-related effects may involve a combination of incomplete epigenetic erasure and RNA-mediated signaling mechanisms, offering a biologically plausible framework despite extensive reprogramming.

Candidate gene bias, limited sample size, tissue specificity, temporal variability, population heterogeneity, and ongoing debate regarding epigenetic reprogramming highlight the need for cautious interpretation. Reported associations between trauma exposure and epigenetic variation should not be equated with definitive evidence of stable transgenerational transmission. Future research will benefit from larger cohorts, epigenome-wide approaches, longitudinal sampling, and integration of molecular, clinical, and environmental data to clarify the scope and mechanisms of multi-generational trauma-related biological embedding.

## 7 Conclusion

**Figure 2:**
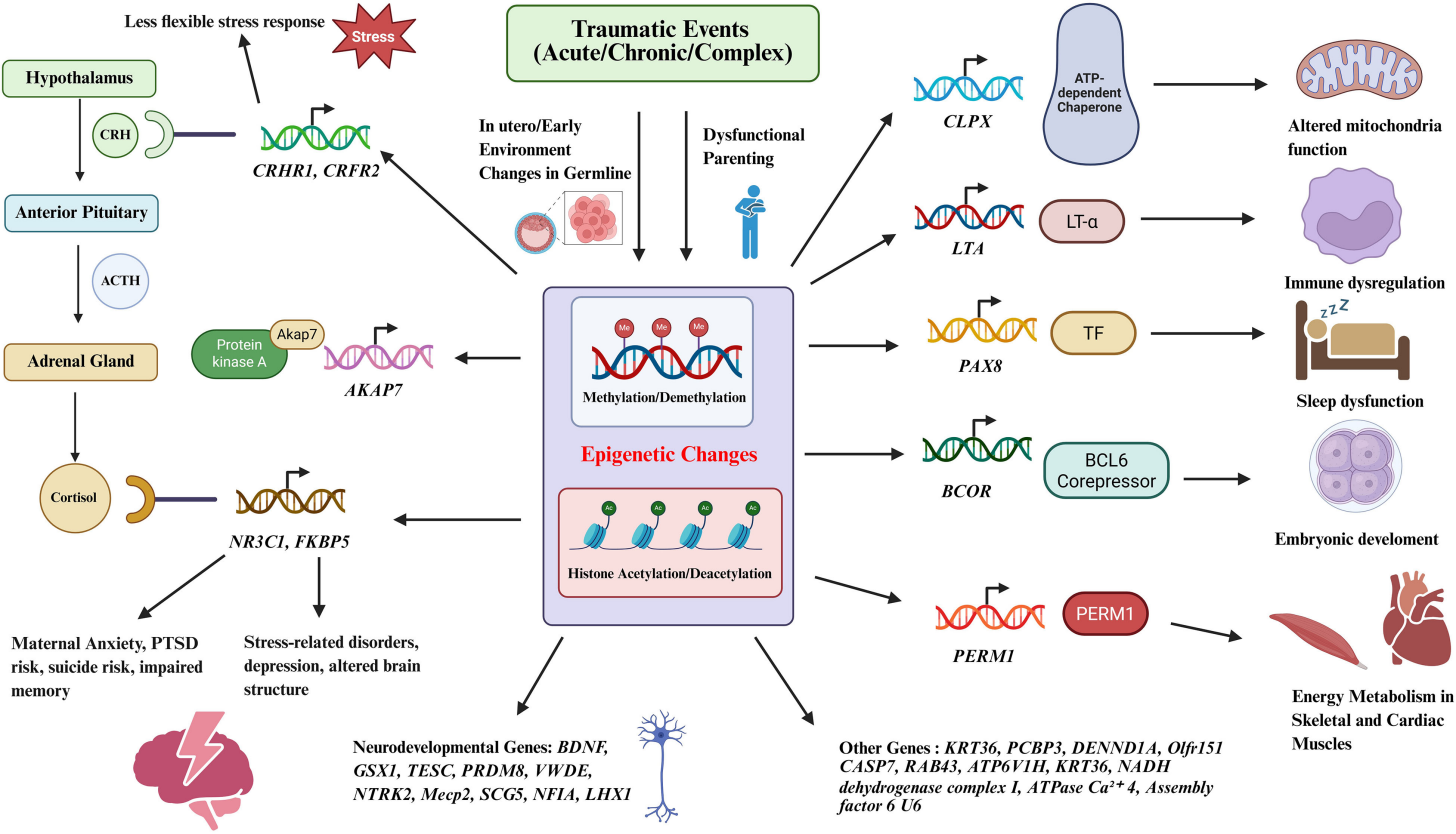
Pathways involved in genes with observed epigenetic changes associated with multi-generational trauma. DNA methylation, histone modification, and noncoding RNA-mediated regulation represent major epigenetic mechanisms reported across trauma contexts. Increased or decreased methylation and/or acetylation of genes involved in the HPA axis, neurodevelopmental pathways, mitochondrial regulation, energy metabolism, and embryonic development have been described in survivors and offspring. These regulatory changes are associated with variation in stress-related, psychiatric, metabolic, and inflammatory phenotypes.

Evidence across acute, chronic, and complex trauma contexts suggests that trauma exposure may be associated with coordinated alterations in stress-response regulation, immune-inflammatory signaling, neurodevelopmental processes, metabolic pathways, and epigenetic remodeling (**Figure 2**). These regulatory changes are frequently described alongside shifts in caregiving behaviors and psychosocial environments, indicating that biological and relational systems may interact in shaping intergenerational vulnerability.

Acute traumatic events, particularly when occurring during pregnancy, are associated with stress-related and inflammatory signaling that may influence fetal developmental programming. Chronic exposures reflect cumulative physiological adaptation, often accompanied by sustained alterations in stress-regulatory and metabolic systems. Complex trauma, characterized by prolonged and severe interpersonal or collective adversity, appears associated with broader regulatory disruption across multiple interacting biological pathways. Across trauma types, offspring outcomes most consistently include increased vulnerability to anxiety, depressive symptoms, stress-related disorders, and certain chronic medical conditions.

Although recurrent findings implicate genes such as *NR3C1* and *FKBP5*, the broader pattern across studies suggests involvement of integrated regulatory networks rather than isolated loci. Trauma-associated epigenetic variation is best interpreted within a systems framework that considers developmental timing, cumulative burden, sociocultural context, and caregiving stability. Current evidence supports association rather than definitive causation, and the persistence, reversibility, and functional significance of reported epigenetic marks remain areas of active investigation.

Important knowledge gaps remain. Larger epigenome-wide studies, longitudinal multi-generational cohorts, and integrated analyses examining parental and offspring biological profiles alongside clearly defined behavioral outcomes are needed. Standardized frameworks for defining intergenerational and transgenerational trauma-related outcomes would also improve cross-study comparability and strengthen inference. In addition, further research is required to determine whether trauma-associated epigenetic variation reflects stable biological embedding, dynamic environmental responsiveness, or a combination of both.

From a therapeutic perspective, existing interventions primarily target psychological symptoms and caregiving environments, with emerging interest in whether trauma-associated epigenetic features may function as biomarkers of vulnerability or treatment responsiveness. At present, evidence does not support direct epigenetic modification as a clinical intervention. Clarifying the role of regulatory and epigenetic variation in risk stratification, recovery, and resilience remains an important future direction.

Overall, the available literature suggests that trauma exposure may relate to coordinated biological and psychosocial processes that extend across generations. Advancing understanding of these interactions will require continued integration of molecular, developmental, clinical, and environmental perspectives. Such work has the potential to refine prevention strategies, inform treatment selection, and improve outcomes for trauma-affected families while maintaining appropriate caution regarding causal inference.
